# Marsh Interspersion and Muskrat (*Ondatra zibethicus*) Habitat Use

**DOI:** 10.1002/ece3.73155

**Published:** 2026-03-02

**Authors:** Gregory P. Melvin, Jeff Bowman

**Affiliations:** ^1^ Environmental & Life Sciences Graduate Program Trent University Peterborough Ontario Canada; ^2^ Wildlife Research and Monitoring Section, Ontario Ministry of Natural Resources Trent University Peterborough Ontario Canada

**Keywords:** camera traps, intensity of use, invasive species, muskrats, *Typha*, wetlands

## Abstract

Muskrat ( *Ondatra zibethicus* ) populations have been declining in North America for decades. The precise cause of these widespread declines has not yet been identified. Over a similar timeframe, wetlands across large regions of North America have been experiencing an invasion of hybrid cattail *Typha x glauca* . This invasion is associated with many negative consequences for wetlands, including a reduction in biodiversity, open water habitat, and interspersion of water and vegetation. Muskrats are strongly tied to wetlands, especially where there is a high degree of interspersion of water and emergent vegetation. Therefore, a widespread reduction in interspersion caused by *T. x glauca* invasions may be contributing to widespread muskrat population declines. We sought to better understand the impact of marsh interspersion on fine‐scale muskrat habitat use in light of widespread invasions of *T. x glauca* . We measured intensity of habitat use by muskrats in a large, *Typha*‐dominated marsh in south‐central Ontario using camera traps, stratifying camera placement along a gradient of interspersion. We found no correlation between interspersion and intensity of use. The ubiquity of *T. x glauca* and low overall interspersion at our study site may have prevented a robust test of our hypothesis. Further research is needed to determine precisely how interspersion affects muskrat habitat use at a fine scale, and how potential changes in habitat quality and use may be contributing to widespread muskrat population declines.

## Graphical abstract

We used camera traps to measure intensity of habitat use by muskrats along a gradient of marsh interspersion to determine whether reduced interspersion, which may be linked to the invasion of *Typha x glauca* , may be contributing to widespread muskrat population declines. Contrary to our prediction, we did not find a correlation between intensity of use and interspersion. More research is needed to determine the cause(s) of widespread muskrat population declines.

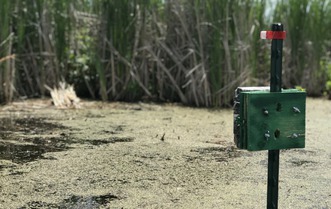

## 1 Introduction

Muskrat harvest has dramatically declined in many North American jurisdictions between the middle‐ to late‐20th century and early 21st century. Analyses of these harvest trends while controlling for pelt prices suggest widespread population declines (Roberts and Crimmins 2010; Ahlers and Heske 2017). Moreover, several empirical studies corroborate these declining trends. For example, muskrat house counts, a proxy for population density, declined by over 90% in two large marshes in southern Ontario, Canada, from the late 1960s to present (Sadowski and Bowman 2021). House counts also declined dramatically in wetlands in northern Alberta, Prince Edward Island, and Connecticut over similar time frames (Ward and Gorelick 2018; Gregory et al. 2019; Benoit and Askins 1999; respectively). These declines are concerning, as muskrats are widely regarded as ecosystem engineers for their activity in marshes (Higgins and Mitsch 2001; Toner et al. 2010; Mott et al. 2013; Kua et al. 2020). For example, depending on the slope and available substrate within the habitat, muskrats either construct houses from emergent vegetation or excavate bank dens (Dozier 1948), creating unique microhabitats that are also used by a multitude of other taxa, including birds, reptiles, and mammals for behaviors such as basking and nesting (reviewed by Kiviat 1978). Muskrats are important marsh herbivores (Errington 1963; Hewitt and Miyanishi 1997) and are known to selectively remove large quantities of emergent vegetation through feeding and creating channels for navigation (Boutin and Birkenholz 1987; Hewitt and Miyanishi 1997; Bomske and Ahlers 2020). These activities, including house‐building, herbivory, and the creation of channels, are associated with increased habitat complexity (Weller and Spatcher 1965; Errington 1963; Wilcox and Meeker 1992; Kua et al. 2020; Lishawa et al. 2025) and biodiversity (Weller and Spatcher 1965; Kaminski and Prince 1981; Nyman et al. 1993; Danell 1996; Kua et al. 2020). Therefore, a decline in muskrats may lead to a decline in habitat structure and biodiversity in wetlands (Baici et al. 2024).

Historically, localized muskrat population declines have been linked to water level fluctuations (Bellrose and Brown 1941; Errington 1951), resource limitations (Dozier 1948; Errington 1963; Weller and Spatcher 1965), disease outbreaks (Errington 1963), and increases in predation (Errington 1939, 1951). However, muskrats are a hardy, resilient, and highly fecund species (Errington 1963; Boutin and Birkenholz 1987; Straka et al. 2018; Kroll and Meeks 1985; Ganoe et al. 2020; Sadowski and Bowman 2021). They can occupy a variety of aquatic habitats (Errington 1963; Virgl and Messier 1997; Ahlers et al. 2010), they can withstand high levels of harvest (Soper 1942; Errington 1951), and local populations are expected to quickly rebound from short‐term die‐offs (Errington 1951; Danell 1978; Kroll and Meeks 1985). Therefore, population declines of the magnitude and duration observed in recent decades are likely the result of a persistent and widespread stressor that is still poorly understood.

Habitat change has been suggested as a plausible mechanism underlying muskrat declines (Ahlers and Heske 2017; Greenhorn et al. 2017; Sadowski and Bowman 2021; Melvin et al. 2024). For example, marsh habitats in large regions of North America have been extensively colonized by invasive cattail (*Typha* spp.; Pieper et al. 2020; Tangen et al. 2022; Stewart et al. 2023; Melvin et al. 2024). Central to *Typha* invasions in North America is *T. x glauca* , a hybrid of native *T. latifolia* and introduced *T. angustifolia* (Smith 2000). Following the gradual spread of *T. angustifolia* and systematic hybridization with its native counterpart, *T. x glauca* has been quick to invade North American wetlands (Galatowitsch et al. 1999) thanks to a suite of competitive advantages and favorable environmental conditions such as high nutrient outputs from agriculture and stabilized water levels resulting from water level management (reviewed by Bansal et al. 2019). The invasion of *T. x glauca* is altering the structure and composition of marshes (Boers et al. 2007; Wilcox et al. 2008; Markle et al. 2018), and the impact on muskrats remains unclear.

The invasion by *T. x glauca* in the Laurentian Great Lakes Region (Pieper et al. 2020; Melvin et al. 2024) and Prairie Potholes Region (Tangen et al. 2022), generally leads to a reduction in marsh interspersion (Wilcox et al. 2008; Schummer et al. 2012; Markle et al. 2018), which may be defined as the degree of intermixing of water and emergent vegetation (Weller and Spatcher 1965; Rehm and Baldassarre 2007; Schummer et al. 2012; Chabot et al. 2014; Melvin et al. 2024). Muskrat populations are positively influenced by marsh interspersion (Weller and Spatcher 1965; Proulx and Gilbert 1983; Melvin et al. 2024). For example, muskrat house density was positively correlated with interspersion in southern Ontario marshes (Melvin et al. 2024). In another southern Ontario marsh, muskrat home ranges always contained a minimum areal coverage of 25% open water and 25% emergent vegetation, while the average home range contained equal proportions of each of these cover types (Proulx and Gilbert 1983). Muskrat population growth was also found to be highest when there was an equal ratio of open water to emergent vegetation present (Weller and Spatcher 1965). Reduced interspersion may result in limited open water travel routes (Greenhorn et al. 2017) and reduced overall habitat connectivity (Sadowski and Bowman 2021) for muskrats. Emergent‐water edges are important habitat features for muskrat house‐building (Sadowski and Bowman 2021) and likely travel, as muskrats tend to avoid deep open water areas absent of emergent vegetation (i.e., ~1.5 m or more; Errington 1963; Ervin 2011; Larreur et al. 2020) and rely on emergent vegetation for cover (Bellrose and Brown 1941). Therefore, evidence suggests that muskrats will be negatively affected by a widespread reduction in marsh interspersion. As invasive macrophytes such as *T. x glauca* reduce interspersion in marshes, muskrats may begin to avoid these degraded habitats, which could ultimately contribute to a decline in muskrat populations.

Based on the findings of previous studies, we hypothesized that muskrats avoid marsh habitat with low interspersion. We conducted a study using camera traps to measure muskrat intensity of use, defined as “the rate of use of a specific resource in a defined unit of time” (Keim et al. 2019), in a large *Typha*‐dominated marsh with a mosaic of varying interspersion. We predicted a positive correlation between muskrat intensity of use and interspersion. The goal of our study was to improve our understanding of the importance of interspersion to muskrat behavior, which may help us understand how muskrats may be vulnerable to habitat changes such as those caused by invasive macrophytes like *T. x glauca* , making the connection between fine‐scale habitat use and widespread population trends.

## 2 Methods

### 2.1 Study Area

**FIGURE 1:**
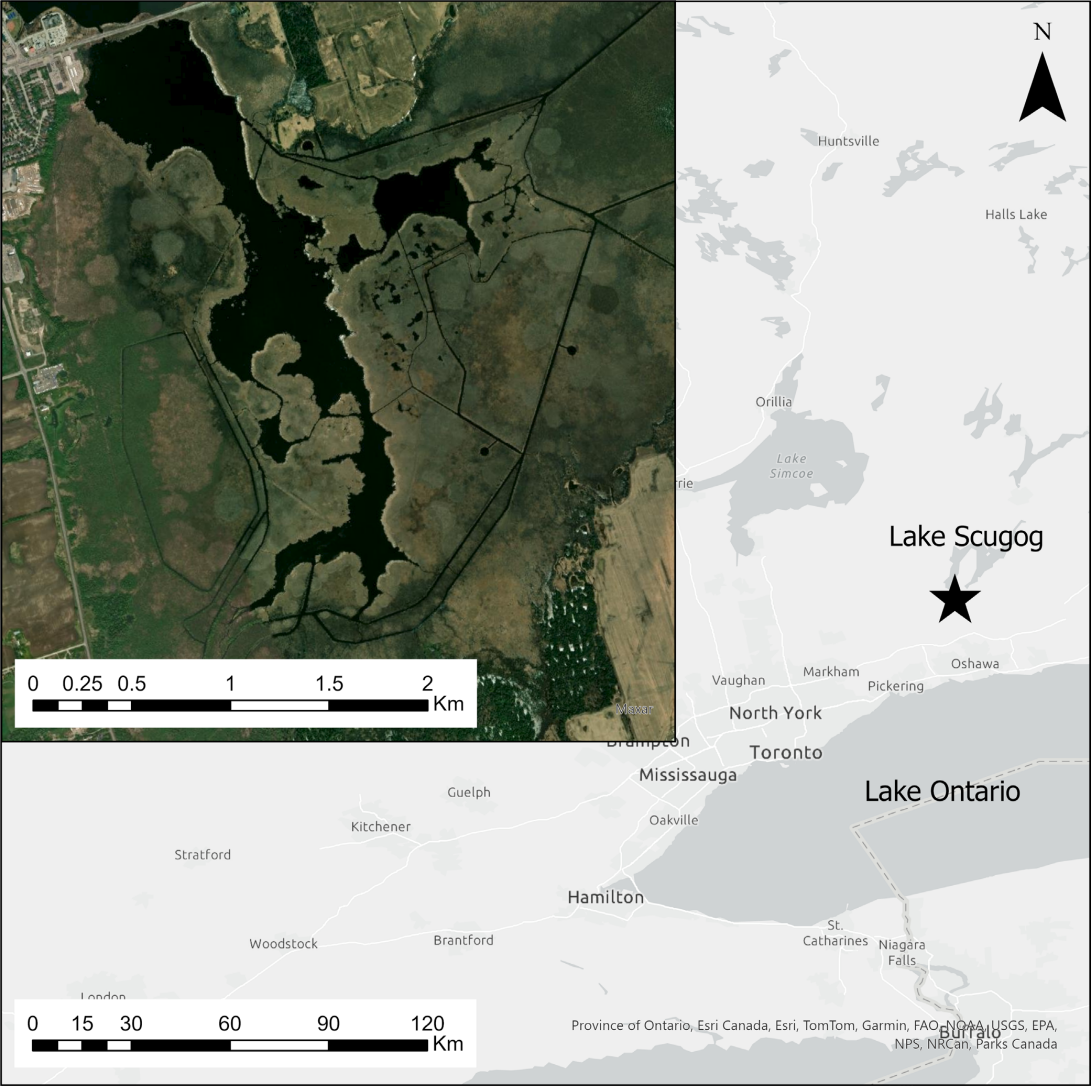
Aerial view of Osler Marsh, Ontario. Marsh location indicated by star on overview map.

The study was conducted from spring to autumn of 2021 at Osler Marsh (44.090° N, 78.920° W) in Scugog Township, Ontario, Canada, at the southern end of Lake Scugog (Figure 1). The marsh occupied roughly 4 km^2^ and was interspersed by patches of open water and manmade channels. The marsh was dominated by invasive hybrid cattail ( *T. x glauca* ), followed by wild rice (*Zizania* sp.) and water lilies (family *Nymphaeceae*). Other prominent species included milfoil (*Myriophyllum* spp.), common duckweed ( *Lemna minor* ), European frogbit ( *Hydrocharis morsus‐ranae* ), ferns (subclass *Polypodiidae*), and various graminoids. To a lesser extent, the marsh contained arrowhead (*Sagittaria* spp.), bulrush (*Schoenoplectus* spp.), alder (*Alnus* sp.), and white cedar ( *Thuja occidentalis* ). The marsh was immediately surrounded by shrub wetland (~80%), lake (~5%), urban development (~5%), and pasture (~5%). The marsh was home to a population of muskrats as determined by recent and ongoing sightings (G.P.M., pers. obs.; Overgoor 2021). The wetland was privately owned, resulting in very little disturbance from boating activity. Access permissions were granted from the land manager. Muskrat trapping had not occurred in the marsh since 1990 (Overgoor 2021). All locations within the marsh were accessed by canoe or small motor craft.

### 2.2 Land Cover Classifications

**FIGURE 2:**
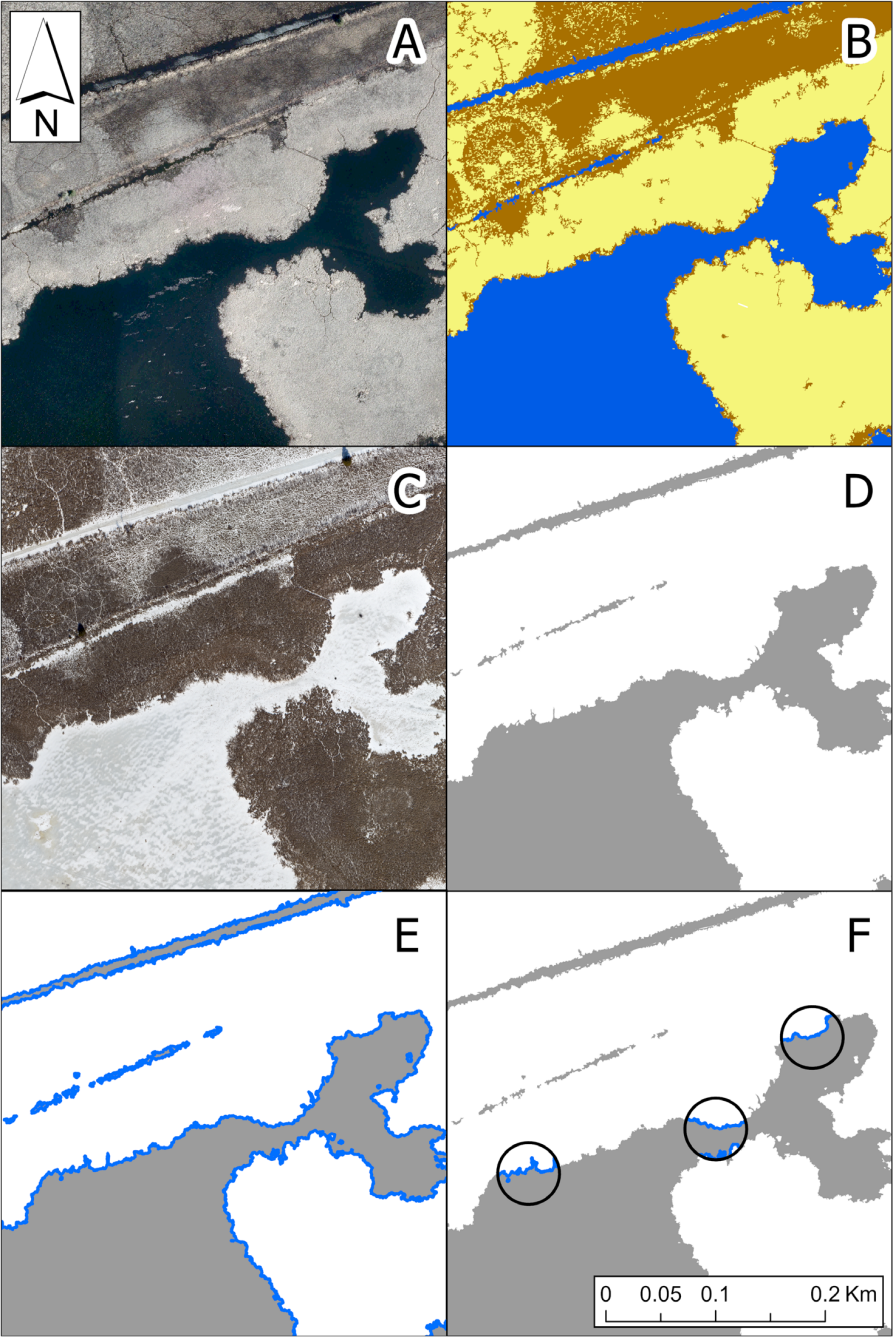
GIS workflow for generating camera trap sample cells. SCOOP 2018 imagery (A) was classified in ArcGIS Pro to identify relevant land cover classes (B; yellow = Cattail, blue = Water, brown = Other) for filtering cells to suitable habitat. Higher resolution imagery collected in 2021 (C) was then classified into two land cover classes (D; gray = Water, white = Vegetation). Water edges (blue lines) were isolated from Water polygons from the previous step to represent emergent‐water edge, i.e., interspersion (E). Water edges were then clipped to quarter‐hectare sample cells (F) and randomly sampled in each sampling period for camera deployment.

We first mapped areas of *Typha*‐dominated vegetation ( *T. latifolia* , *T. angustifolia* , and *T. x glauca* ), open water, and other vegetation (e.g., other emergent aquatic vegetation as well as grasses, forbs, shrubs, and trees), hereafter *Cattail*, *Water*, and *Other*, respectively, so that we could filter our sampling efforts to cattail‐dominated marsh habitat (Figure 2). We produced a map by classifying land cover elements using the Classification Wizard in ArcGIS Pro 2.6 (Environmental Systems Research Institute 2021) along with aerial imagery from the South‐Central Ontario Orthophotography Project 2018 (SCOOP 2018) collected by Land Information Ontario (Land Information Ontario [LIO] 2019) to identify the three habitat classes (*Cattail*, *Water*, and *Other*). Collected in spring 2018 and 2019, SCOOP 2018 was the most recent high‐resolution imagery available that showed relatively high contrast between *Typha* stands and other land cover types, especially water, and the high (16 cm) resolution allowed us to differentiate habitat classes at a fine scale (i.e., within 0.25 ha sample cells).

### 2.3 Measuring Interspersion

While SCOOP 2018 imagery accurately represented land cover classes throughout the marsh at the time of the study in 2021 (confirmed by ground‐truthing), subtle changes in marsh structure from 2018 to 2021 due to shifting *Typha* mats would have led to spatiotemporally inaccurate measurements of interspersion using SCOOP 2018 imagery. Therefore, to measure interspersion, we classified more recent (winter 2021) imagery collected by the Ontario Ministry of Natural Resources (MNR; outlined by Melvin et al. 2024) to make our measurements of interspersion as temporally relevant as possible; the winter 2021 imagery showed little contrast between *Typha* and other vegetation, and thus was only used to measure interspersion and not to differentiate among vegetation types as we did in the previous step with SCOOP 2018 imagery. The newer imagery was collected at a spatial resolution of approximately 7.5 cm. Snow cover during this time was minimal, and larger (≥ 50 m^2^) snow patches represented water features which we confirmed using SCOOP 2018 which was collected in the spring and contained only unfrozen water. We only classified water and vegetation (i.e., two land cover types) using this imagery which allowed us to measure interspersion (Figure 2). See Appendix 1 for further details on land cover classification methods.

**FIGURE 3:**
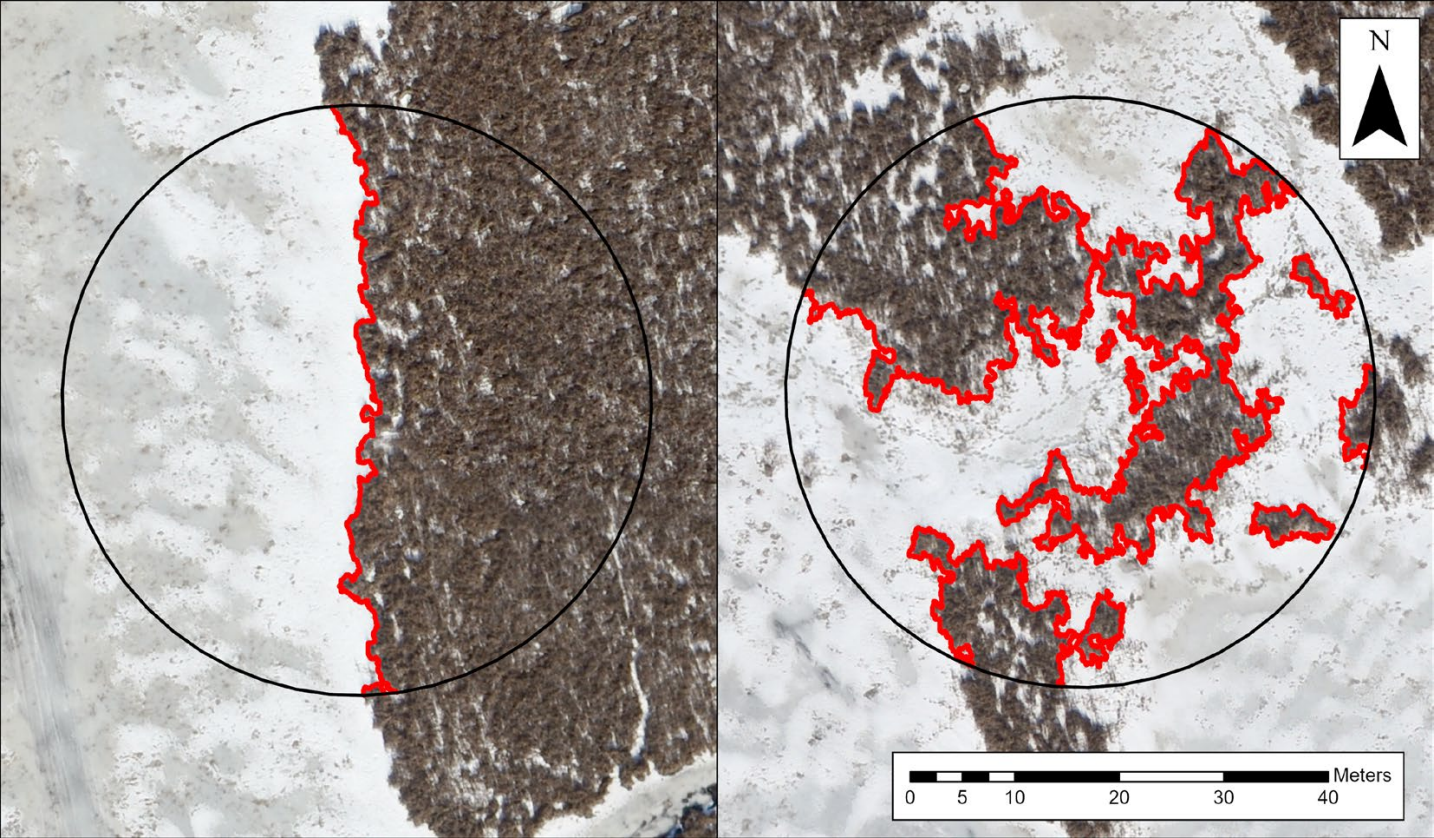
Example quarter‐hectare sample cells with low interspersion (left) and high interspersion (right). Emergent‐water edges used for calculations of interspersion are shown in red. Imagery: Melvin et al. (2024).

We measured interspersion as emergent‐water edge density as has been done by others (Rehm and Baldassarre 2007; Chabot et al. 2014; Hohman et al. 2021; Melvin et al. 2024; Figure 3). Interspersion changes throughout the growing season, and thus, capturing and incorporating these changes into our study design would have been very challenging due to the limited availability of real‐time, high‐resolution imagery, and the time required to classify imagery. Therefore, for simplicity, we used only one set of imagery from the winter prior to our study and one corresponding image classification to assess interspersion, as outlined in the previous paragraph. By doing so, we chose to focus on larger‐scale patterns of interspersion, particularly those that can be observed during the nongrowing season (i.e., standing, senesced plant matter that persists from year to year).

The interface between water features and *Typha*‐dominated marsh was well‐defined, and thus, accurately represented interspersion. However, wind‐blown cattail and shorter vegetation types allowed for buildup of snow drifts within vegetation stands, creating sizeable snow patches that did not represent open water features. This led to inflated estimates of water area, and consequently, interspersion, which was evident when comparing with SCOOP 2018. To avoid snow patches being misclassified as water features and ultimately used in measurements of interspersion, we measured interspersion using only *Water* features ≥ 50 m^2^ within sample cells. This minimum threshold mostly limited our measurements of interspersion to the edges of the main (i.e., largest) water features within sample cells, rather than smaller pools and rivulets. These larger water features are likely the most relevant to muskrat habitat as they would provide easier swimming conditions compared to small rivulets and pools of water hidden among tall, densely packed, robust emergent plants such as *T. x glauca* which largely comprised the interior vegetation stands in the study area. Furthermore, muskrats typically build their houses along the edges of open water (i.e., larger water features) and emergent vegetation (Sadowski and Bowman 2021). Therefore, we used this rationale to measure interspersion using only these larger water features. We thus converted *Water* polygons ≥ 50 m^2^ to lines which represented emergent‐water edges (i.e., interspersion of main water features, hereafter interspersion). In only one sample cell, interspersion still appeared to be overestimated since the outermost vegetation edge contained less cattail and thus did not form a defined boundary with the adjacent pond. To correct for this, we digitized (i.e., manually traced) the portion of the emergent‐water edge where interspersion appeared to be overestimated using SCOOP 2018 as a guide, ensuring that no major shifts in vegetation structure had occurred since the time of acquisition.

### 2.4 Sampling

Cameras were deployed monthly in 0.25 ha sample cells which represented the home range size of muskrats in northern marshes (Takos 1947; MacArthur 1980; Proulx and Gilbert 1983). While home range sizes can vary, and specific home ranges of muskrats within the study area were not established, we used this mean home range size in an effort to make sample cells ecologically relevant for muskrats while also ensuring that enough sample cells could be generated within the study area. To create sample cells, we used ArcGIS Pro to trace lines along the main emergent‐water edges of the marsh, adding points at 60 m increments. These points served as cell centroids around which we created circular 0.25 ha buffers. Interspersion was then calculated for each 0.25 ha cell by intersecting emergent‐water edges with sample cells (Figure 3). We also intersected land cover polygons (derived from SCOOP 2018 imagery) with sample cells to calculate the proportion of each land cover type per cell. We eliminated any cells that contained < 5% *Cattail* and < 5% *Water* by area, as well as cells that contained ≥ 1500 m^2^ of *Other* to ensure that *Typha* was a prominent part of the vegetation community. We sampled 20 cells per monthly sampling period, and each sampling period included similar ranges of interspersion from low (116 m) to high (1094 m). Due to a limited selection of suitable sample cells in the study area by the fourth sampling period, we expanded our selection of available sample cells to include cells which included less open water; in doing so, many of the randomly sampled cells in the fourth sampling period were comprised of narrow channels. In an effort to maintain independence of sample cells throughout the study, we ensured that cells did not overlap within sampling periods, maintaining a minimum Euclidean distance of 75 m between centroids and 15 m between cell edges.

### 2.5 Camera Deployment

**FIGURE 4:**
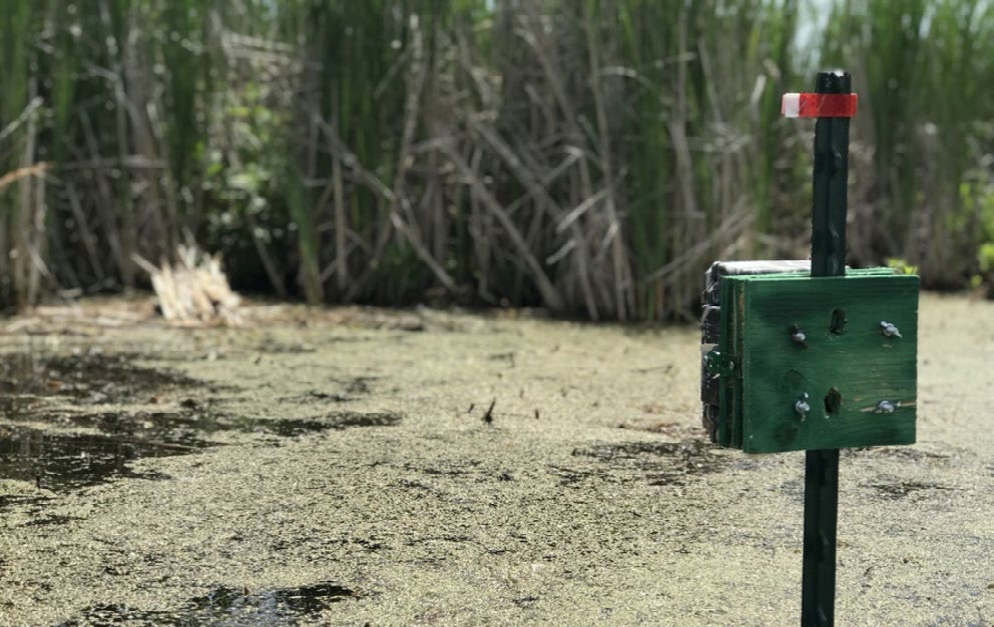
Typical camera configuration.

We positioned unbaited plywood platforms covered with senesced cattail stalks at the centroid of each sample cell along the cattail‐water interface to provide a perch for passing muskrats. Cameras were fixed to iron T‐posts which were inserted into the marsh substrate in open water facing the platform approximately 5 m away (Figure 4). Camera height was adjusted to sit approximately 0.75 m from the water's surface which provided an adequate extent for viewing of muskrats throughout the surface of the water and prevented cameras from being submerged in the event of increased water levels. We oriented cameras to face roughly north or south to avoid glare from direct sunlight. We lightly cleared emergent vegetation within the detection zone to improve visibility; while this may have had a small effect on muskrat activity, it did not affect our measurements of interspersion which focused on larger scale patterns in the landscape and were measured before the growing season.

We used Reconyx Hyperfire 1, Hyperfire 2, and Ultrafire camera traps which were loaded with 16 GB SD cards and Energizer Ultimate Lithium AA batteries. Since muskrats are largely nocturnal (Butler 1885; Chatterton 1944; Dozier 1948; Boutin and Birkenholz 1987; Marinelli and Messier 1993), and semi‐aquatic mammals may not trigger passive infrared sensors at night (Lerone et al. 2015), we did not use motion detection in anticipation of missing all or most passing muskrats. We initially tested this idea by running a 1 month pilot study in April and May, 2021, placing 20 cameras throughout the marsh according to the random cell selection outlined above, with cameras set to record motion‐triggered images at high sensitivity at all times of the day and night. After looking through all photos from multiple cameras, we determined that nighttime muskrat detections were extremely rare, as predicted, even though muskrats are largely nocturnal. Therefore, we adapted the protocol and programmed cameras to take one photo every 5 min from 1600 h to 0900 h. In doing so, we standardized camera sampling effort across sample cells, eliminated the potential for excessive false detections (e.g., due to moving vegetation), and improved the probability of detecting muskrats during crepuscular and nocturnal periods when they are more likely to be active.

**FIGURE 5:**
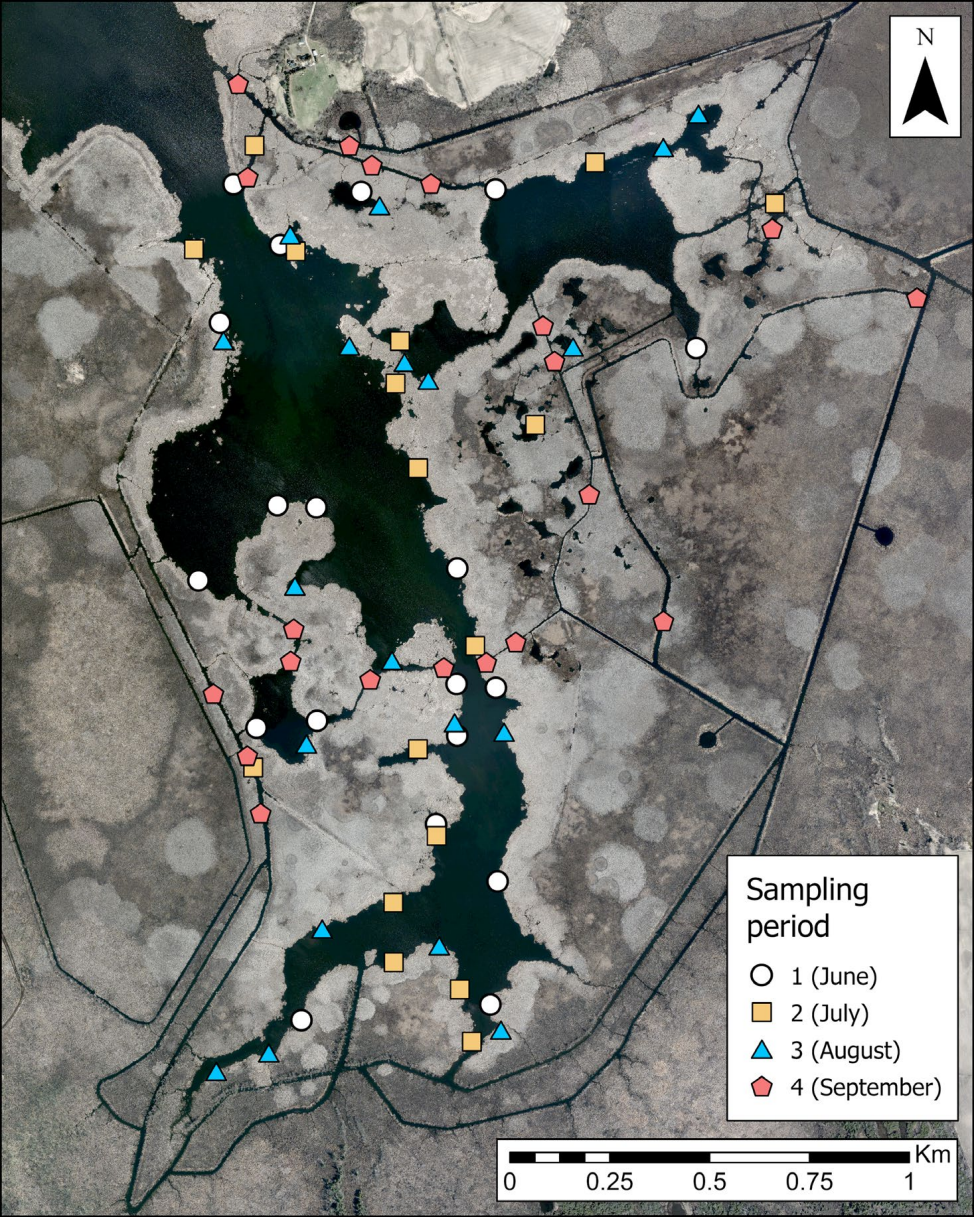
Camera locations at Osler Marsh, Ontario, during each sampling period.

Cameras were first deployed at the end of May 2021 and were moved to new locations after approximately 1 month (average of 28 days) until the final deployment ending in September 2021 for a total of four sampling periods (Figure 5). Sample cells were accessed by watercraft. We could not always exactly locate the cell centroid in the field due to wind gusts displacing our watercraft and certain points lying in very shallow water and mucky substrate that could not be accessed. If we could not deploy a camera and platform at the designated location, we deployed them at the nearest possible location along the cattail‐water edge. We then obtained GPS coordinates at the new platform location and recalculated interspersion and land cover proportions.

### 2.6 Image Tagging and Analysis

One of us (G.P.M.) and a group of trained technicians tagged images using Timelapse 2.0 image tagging software (Greenberg 2022). Due to resemblance with beavers, muskrats were only tagged if the observer was highly confident of species identification based on a set of visual characteristics (e.g., laterally compressed tail, body and tail visible above water's surface, comparatively small body size). We measured the intensity of use as the number of muskrat events per sampling period, and we defined an event as a collection of consecutive muskrat sightings (i.e., 5 min apart). If more than one muskrat could clearly be differentiated within the viewshed, each muskrat counted toward a separate event. It is possible that consecutive photos included a different muskrat in each photo, and therefore should have been classified as separate events. However, most events consisted of only a single photo, and very few events spanned more than two consecutive photos (i.e., ≥ 10 min). To account for potential detection bias due to differences in habitat structure among camera locations, we assigned an index for both sample area (i.e., the water surface in which muskrats could be observed) and viewshed obstruction (i.e., from vegetation) to each camera location to be used as variables in our data analysis. The index for sample area was calculated by estimating the relative difference in the extent of the water surface observed in the viewshed and a standard viewshed in which the water surface occupied roughly half of the viewshed; 1 represented the same water surface extent as the standard viewshed, 0.5 represented half the extent, and 2 represented double the extent. Viewshed obstruction from vegetation was estimated using a ranked scale of 0–4 from lowest to greatest obstruction based on photos from midnight on the same day near the middle of each sampling period (Table A1 in Appendix 1). Since it took multiple days to move cameras to new locations and we could not visit the field site on the same days every month, sampling periods and camera active periods within them each spanned a different length of time. Therefore, we standardized the data by restricting each sampling period to 20 trap nights (i.e., 1600 to 900 h) from the 1st to the 20th of each month which encompassed all camera‐active periods in each sampling period except for cameras whose batteries died prematurely (*n* = 8) and cameras that were programmed incorrectly leading to detection bias (*n* = 5); data from these latter cameras were omitted from our analysis. Sample size ultimately ranged from 17 to 18 camera locations per month.

### 2.7 Data Analysis

We used generalized linear models to evaluate intensity of use by muskrats at Osler Marsh (number of muskrat events per sampling period per location). Variables in the model included interspersion (in meters), as well as several additional variables which may also potentially influence intensity of use for muskrats. Additional variables included the estimated indices of sample area (a continuous index varying between 0.4 and 2.0) and viewshed obstruction (a ranked scale varying between 0 and 4), both of which have the potential to affect detectability of muskrats by observers, while heavy vegetation cover may also impede muskrats from occupying the area. We included the continuous variable of water area (m^2^) within the sample cell (calculated from classification of SCOOP 2018 imagery) which is likely an important determinant of muskrat habitat use (Proulx and Gilbert 1983). We included the binary variable of channelization (channelized vs. non‐channelized water features), since channelized water features where travel routes are constricted may see higher occurrences of muskrats than in less constricted habitats (Pelletier et al. 2014). Sample cells whose main water feature was bordered by vegetation on opposite sides (up to 30 m between sides) and where water connectivity remained continuous at both ends beyond the cell boundaries were considered channelized and were denoted with a value of 1; all other sample cells were considered non‐channelized and were denoted with a value of 0. Finally, we included the variable of sampling period (a ranked scale varying between 1 and 4), as the muskrat activity may be seasonal (MacArthur 1980), with increased activity during periods of house‐building (Errington 1963; Proulx and Gilbert 1984). Since cameras were moved to new locations in each new sampling period, the variable of sampling period also incorporates the effect of camera placement in these new locations during each of the periods. We first used Pearson correlations to explore correlations between intensity of use and each predictor variable. We then used zero‐inflated negative binomial models since our response variable consisted of counts (i.e., muskrat events), contained excess zeros, and the variance was larger than the mean (Stoklosa et al. 2022). To test our prediction that muskrat intensity of use was positively correlated with interspersion, and to evaluate whether the additional variables were stronger predictors, we compared a global model of intensity of use, all possible two‐variable models including interspersion, all possible single variable models, and the null model using Akaike's Information Criterion (AIC) which ranks models from best to worst fit (Burnham and Anderson 2004).

## 3 Results

### 3.1 Overview

From 1 June to 20 September 2021, we detected 156 muskrat events across 69 locations with a mean of 2.26 (±0.47 SE) events per location. Muskrats were detected at 38 locations (55%). The greatest intensity of use during any sampling period and at any location was 19 events. An additional 71 events in which unknown rodents could not be confidently identified as muskrats were not included in our analyses.

### 3.2 Pearson Correlation

**FIGURE 6:**
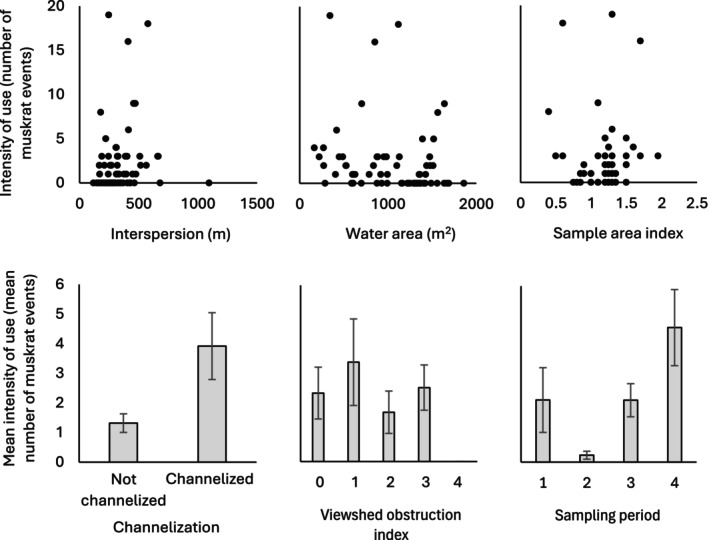
Intensity of use by muskrats and six predictor variables. See methods for definitions of each variable.

We did not find a strong correlation between interspersion and intensity of use (*r* = 0.13, *p* = 0.28; Figure 6). Intensity of use was positively correlated with channelization (*r* = 0.32, *p* = 0.0074) with greater muskrat activity in channelized (*x̄* = 3.9 ± 1.13 SE) than in non‐channelized habitat (*x̄* = 1.32 ± 0.31 SE). Intensity of use was also positively correlated with sampling period and location (*r* = 0.26, *p* = 0.029) and was highest in September (*x̄* = 4.6 events per location ±1.30 SE). We observed a small, negative correlation between intensity of use and water area in the sample cell (*r* = −0.20, *p* = 0.11). We did not observe strong correlations between intensity of use and sample area (*r* = 0.023, *p* = 0.85) or viewshed obstruction (*r* = −0.088, *p* = 0.47). Correlations between all pairs of predictor variables were below 0.7 (or above −0.7) and thus were not strongly correlated according to Ratner (2009).

### 3.3 Modeling

When comparing all models using AIC, the global model had the best fit, while other models containing interspersion did not perform as well as other single‐variable models (Table 1). Channelization and water area were the only significant predictors of intensity of use in the count portion of the global model (Table 2). There were no significant predictors of excess zeros in the global model. Other models of intensity of use can be found in Table A2 in Appendix 1.

**TABLE 1:** Akaike's information criterion values for thirteen models of muskrat intensity of use at Osler Marsh, Ontario, in decreasing order of support.

Model	df	AIC	ΔAIC
Interspersion + Sampling period + Water area + Channelization + Viewshed obstruction + Sample area	15	262.96	
Channelization	5	268.26	5.30
Sampling period	5	268.76	5.80
Water area	5	269.41	6.45
Interspersion + Channelization	7	269.94	6.98
Viewshed obstruction	5	271.25	8.29
Interspersion + Water area	7	271.29	8.33
Interspersion + Sampling period	7	271.51	8.55
Null	3	272.27	9.31
Interspersion	5	273.18	10.22
Interspersion + Viewshed obstruction	7	274.65	11.69
Sample area	5	275.98	13.02
Interspersion + Sample area	7	276.60	13.64

**TABLE 2:** Summary of the strength and direction of the relationship between six predictor variables and intensity of use by muskrats at Osler Marsh, Ontario, as determined from a zero‐inflated negative binomial model (global model of intensity of use). Significant predictors are indicated in bold text.

Variable	Estimate	Std. error	*z*‐value	*p*
Count model				
Channelization	**1.76**	**0.447**	**3.94**	**8.13e** ^**−5**^
Water area	**1.77e** ^**−3**^	**6.92e** ^**−4**^	**2.56**	**0.0104**
Sampling period	0.446	0.251	1.78	0.0758
Sample area	−0.660	0.602	−1.097	0.273
Interspersion	−4.76e^−4^	1.21e^−3^	−0.395	0.693
Viewshed obstruction	−0.0851	0.219	−0.389	0.697
Zero‐inflation model				
Interspersion	−0.010	6.06e^−3^	−1.70	0.0893
Channelization	3.33	2.34	1.43	0.154
Viewshed obstruction	1.44	1.11	1.30	0.193
Sampling period	−0.829	0.753	−1.10	0.271
Water area	3.39e^−3^	3.29e^−3^	1.03	0.303
Sample area	−0.316	2.25	−0.140	0.889

## 4 Discussion

Contrary to our prediction, we did not find that muskrat intensity of habitat use was correlated with interspersion at Osler Marsh. Moreover, aside from the global model of intensity of use, models containing interspersion generally did not perform well. Intensity of use was more heavily influenced by other factors, including channelization, sampling period and location, and water area. The lack of correlation between interspersion and intensity of use may be due to site‐specific habitat features which acted more strongly on intensity of use, but our methodology may have also had an impact on our results, preventing a rigorous test of our hypothesis. For example, *T. x glauca* was widespread at our study site and variation in interspersion may have been insufficient to detect an effect on muskrat intensity of use.

Intensity of use was most strongly correlated with channelization, with greater use of channelized over non‐channelized habitats. Channels were dredged at Osler Marsh for boat travel nearly two centuries ago (Hvidsten 2017). These dredged channels and other naturally occurring channels within the marsh were mostly narrow (approximately 3–8 m wide, but some as wide as 30 m), sheltered, and relatively still compared to the large ponds central to the marsh which frequently experienced strong wind gusts (G.P.M., pers. obs.). Muskrats typically avoid traveling across large, exposed water bodies (e.g., lakes; Ervin 2011; Larreur et al. 2020), thereby making these channels potentially attractive to muskrats. Most channels at Osler Marsh were still cleared of vegetation annually using a barge with a mechanical tiller for ease of navigation, likely facilitating travel for muskrats as well, compared to pond edges where other cameras were located which became thick with wild rice and lilies over the summer. Finally, since these channels were relatively narrow, on average, and muskrats avoid traveling out of water (Ahlers et al. 2010), channels may also act as constricted travel corridors, thereby increasing muskrat traffic. Therefore, muskrats at Osler Marsh may select channelized habitat more strongly than highly interspersed habitat, but it may also be more likely to observe muskrats on camera in these high‐traffic areas where muskrat activity is more concentrated. However, channels at Osler Marsh may provide similar functions as highly interspersed habitat, such as increased ease of movement relative to denser stands of cattail. One study showed that the creation of hemi‐marsh conditions (i.e., maximum interspersion) through mechanical methods maximized densities of several species of dabbling ducks (Masto et al. 2022), whose habitat requirements overlap considerably with muskrats (Weller and Spatcher 1965; Bishop et al. 1979). Therefore, as suggested by our results, mechanically clearing invasive plants may have the potential to improve habitat conditions for muskrats, though this hypothesis will require further empirical testing.

Due to a limited selection of suitable sample cells remaining by the fourth sampling period, we included mostly channelized habitat in this period, potentially confounding the effects of channelization with the effects of seasonality. Muskrat density likely increases at Osler Marsh through the summer as new litters are produced (Boutin and Birkenholz 1987), which may explain the positive correlation between intensity of use and sampling period. However, channelized habitat had a larger effect on muskrat intensity of use than sampling period as determined by our global model (Table 1). Limiting cell selection to a single habitat type (i.e., channelized or nonchannelized) would improve inferences of intensity of use in relation to interspersion in future studies.

The negative correlation between water area and intensity of use in this study is somewhat surprising, but can likely be attributed to the negative correlation between water area and channelized habitat, as channelized habitats generally contained less open water relative to emergent vegetation. Therefore, greater intensity of use in channelized habitats may indicate the prioritization of channelized habitat over water area. However, muskrat traffic in front of these cameras due to the confined swimming space within these channels may have also been higher, as previously mentioned, leading to higher estimates of intensity of use.

The scale at which interspersion is important to muskrats is not widely reported in the literature. Proulx and Gilbert (1983) found high levels of interspersion within muskrat home ranges in a southern Ontario marsh, ranging from 484 m^2^ in early summer to 1112 m^2^ in late summer. Melvin et al. (2024) showed that interspersion within 1 ha grid cells, averaged within 39 southern Ontario marshes, was a significant predictor of muskrat population density. Since we measured interspersion at a scale of 0.25 ha sample cells (2500 m^2^), it is likely that we appropriately captured the scale at which interspersion is relevant to muskrats. However, it is possible that changing the scale at which interspersion was measured may have led to different results. For example, site‐level interspersion at Osler Marsh, calculated in meters per hectare and averaged across the marsh, was found to be low (263 m/ha) near the time of this study compared to other marshes in southern Ontario (Melvin 2023). Low interspersion at the marsh level may have led to reduced variance in interspersion across our sample cells, thereby limiting a robust test of our hypothesis. Determining the optimal resolution at which to measure interspersion may thus be important for future studies examining interspersion and muskrat habitat use.

While the average muskrat home range may be roughly 0.25 ha, home range size can vary widely, from a radius of as little as 15 m (MacArthur 1978) to 230 m (MacArthur 1980) from a dwelling. Studies suggest that muskrat home range size is negatively correlated with population density (Proulx and Gilbert 1983; Marinelli and Messier 1993), as seen in other rodents (Maza et al. 1973; Cameron and Spencer 1985; Erlinge et al. 1990). Muskrat house density at Osler Marsh near the time this study was conducted was low compared to other marshes in south‐central Ontario (Melvin 2023), which were also low, on average, compared to historical house densities in *Typha*‐dominated marshes in Canada (Proulx and Gilbert 1984; Messier and Virgl 1992; Sadowski and Bowman 2021). This may have led to relatively large home range sizes at Osler Marsh, and thus, it is possible that we did not capture entire home ranges in our 0.25 ha (~28 m radius) sample cells. We also assumed equal home range configurations throughout the marsh, though evidence suggests that linear water features may lead to increased length of home range size relative to nonlinear features (Ahlers et al. 2010). This may have resulted in home ranges of muskrats that spanned multiple sample cells in the marsh. One may expect fewer passes in front of a given camera in home ranges spanning multiple cameras, though this may be compensated by increased movement of individuals that is typical in larger home ranges (Alt et al. 1980; Gehring and Swihart 2004; Keim et al. 2019). Furthermore, muskrat home ranges may overlap (Marinelli and Messier 1993; Ganoe et al. 2021), resulting in potentially more than one home range occurring within our sample cells. Therefore, though it may be difficult to adequately account for muskrat home range, home range is unlikely to be a critical factor in determining the size of sample cells when studying intensity of use in muskrats.

Classifying imagery acquired before each sampling period and including all vegetation, living and dead, would likely have led to different measurements of interspersion compared to the nongrowing season. However, Melvin et al. (2024) found that muskrat house densities were highest, on average, at sites with high interspersion, where interspersion was measured using imagery captured in spring (SCOOP 2018) which only captured senesced *Typha* and vegetation stands which tend to persist from year to year and are much less dynamic than summer growth that occurs outside of these stands. This suggests that these persistent vegetation stands may be stronger driving forces acting on muskrat abundance and distribution than spring and summer growth. We effectively employed the same technique by using imagery captured during the non‐growing season (i.e., winter). To our knowledge, no previous studies have used winter imagery to assess interspersion in a marsh. Other methods of assessing interspersion, such as using imagery collected in a different season or at a different resolution, or measuring interspersion at a different scale, may have produced different results. However, for the purposes of our study, we believe that we appropriately measured interspersion in the context of muskrat ecology.

Daily activity patterns of muskrats at Osler Marsh were not determined prior to conducting our study. Therefore, measuring intensity of use using only observations from 1600 to 0900 h may have unintentionally biased our results, since muskrats could be more active during the day than at night, on average, at our study site. Indeed, some muskrats were detected between the hours of 0900 and 1600 h via motion detection during our pilot study; however, these were rare events compared to average monthly detection rates from 1600 to 0900 h during our formal study. Nevertheless, monitoring muskrat activity for the full 24 h period would have eliminated this potential bias, with the trade‐off of having considerably more photos to process. Furthermore, using only interval‐timer photos instead of motion‐detection photos would have led to missed muskrat detections across locations, and this may have altered relative estimates of intensity of use across locations. However, restricting sampling to interval time photos yields standardized and comparable sampling of intensity of use across locations. Conversely, using motion‐detection introduces its own set of problems, including excessive non‐target photos (e.g., due to moving vegetation or abundant non‐target species) and, in the case of small‐bodied, swimming mammals that may be nocturnal, such as muskrats, there may be a complete lack of detections at night, thereby introducing a different detection bias. As camera trap technology improves, these problems will be minimized.

In addition to having low site‐level interspersion, Melvin (2023) confirmed that Osler marsh was dominated by *T. x glauca* at the time of this study. However, without baseline data for comparison, we cannot be certain that the low interspersion at this site was caused by the invasion of *T. x glauca* . Since the marsh was dominated by *T. x glauca* , we did not conduct any statistical comparisons between the distribution of *T. x glauca* and intensity of use by muskrats. Regardless, *Typha* sampling and subsequent identification could only be conducted later in the summer during the pollen‐shedding and flowering periods which occur in early to mid‐summer in southern Ontario (Ball and Freeland 2013), and thus, we were unable to incorporate the distribution of *T. x glauca* and native *T. latifolia* into our study design, focusing instead on the effects of interspersion on intensity of use. Melvin et al. (2024) found little correlation between *T. x glauca* relative abundance and muskrat population density; however, that inference was similarly limited by the high degree of dominance by *T. x glauca* across sites. A study of muskrat habitat use stratified by *Typha* taxa in a wetland with a highly variable cattail community would likely provide further insight into the effects of *T. x glauca* invasions on muskrats, though such wetlands may be difficult to find.

## 5 Conclusions

We did not find that interspersion was an important predictor of intensity of use by muskrats at Osler Marsh. However, our study design may have led to limitations which precluded a rigorous test of our hypothesis. The scale and resolution at which interspersion is measured may be important considerations for future fine‐scale studies of muskrat habitat use. Identifying a gradient in *T. x glauca* in advance would also be beneficial. Improving estimates of intensity of use by fully capturing activity patterns will also likely yield more robust results. Nevertheless, intensity of use appeared to be strongly influenced by channelized water features. The frequent use of channels by muskrats in our study suggests that mechanical control of invasive wetland plants might successfully be employed to recover muskrat populations, but further empirical testing is necessary. Despite our results, interspersion is an important habitat feature for muskrats, but this may not be reflected in studies of intensity of use, and thus, intensity of use may not be the best metric to predict widespread population declines. While there remains little empirical evidence directly linking reduced interspersion and invasive species with muskrat population declines, the continued spread of *T. x glauca* and associated changes to muskrat habitat are difficult to ignore. We recommend that agencies continue to monitor marsh communities and muskrat populations across the muskrat's native range, as well as further targeted studies to adequately test whether wetland invasions and associated habitat changes are leading to muskrat population declines.

## Author Contributions

**Gregory P. Melvin:** conceptualization (equal), data curation (lead), formal analysis (lead), investigation (equal), methodology (lead), project administration (equal), resources (lead), visualization (lead), writing – original draft (lead), writing – review and editing (supporting). **Jeff Bowman:** conceptualization (equal), data curation (supporting), formal analysis (supporting), funding acquisition (lead), investigation (supporting), methodology (supporting), project administration (equal), resources (supporting), supervision (lead), writing – original draft (supporting), writing – review and editing (lead).

## Funding

This work was supported by the Ministry of Natural Resources. Natural Sciences and Engineering Research Council of Canada.

## Conflicts of Interest

The authors declare no conflicts of interest.

## Data Availability

Data are archived in the Dryad data repository at: https://doi.org/10.5061/dryad.866t1g1wp.

## Appendix 1

### Land Cover Classifications

We used an object‐based classification to identify land cover types, which is considered superior to pixel‐based classification for high‐resolution imagery of wetlands (Grenier et al. 2008; Luymes and Chow‐Fraser 2021), and we used an unsupervised classification approach as we did not collect a robust random sample of training data generally required for supervised classification (Lu and Weng 2007; Hu et al. 2017). As part of the object‐based classification process, we created a segmented image using a spatial detail of 20, spectral detail of 20, and minimum pixel size of 30; after adjusting settings and running multiple iterations, these parameters best represented the observed habitat classes within the marsh overall. We defined *Cattail* as emergent vegetation stands containing at least 50% *Typha* relative to other vegetation types. Spectral overlap between certain features (e.g., dense cattail stands and water sheen which may both appear white) caused some misclassifications. We visually compared the classified raster to the imagery and manually reclassified features that were clearly misclassified. We then converted the raster to polygons representing each class (*Cattail*, *Water*, and *Other*) for ease of spatial processing. Finally, we applied two additional steps to improve classification accuracy. First, to reduce speckle (i.e., misclassified pixels or groups of pixels due to high heterogeneity among neighboring pixels), we converted all *Other* polygons < 5 m^2^ to *Cattail*; most of these polygons represented cattail shadows, and this reclassification had no perceptible effect on the representation of actual vegetation features in the *Other* class. Secondly, we corrected for major temporal differences in marsh structure by digitizing (i.e., manually tracing) major changes in marsh structure from 2018 (SCOOP 2018 imagery) to 2021 (imagery acquired by the Ontario Ministry of Natural Resources [MNR]) and incorporating these changes into our final land cover classification. We used 167 ground‐truth points to verify the accuracy of our land cover classification. A minimum of 46 points per class were opportunistically sampled at the marsh throughout 2021. GPS locations were collected with a Garmin handheld GPS unit or iPhone SE 1st generation with ≤ 5 m accuracy. Some ground‐truth points were not accessed directly but examined from the water's edge; these points were moved approximately 5 m inland from the water's edge prior to the accuracy assessment, representing the area observed by surveyors. We computed an accuracy assessment using a confusion matrix, resulting in an overall accuracy of 82.0%. Like our classification of SCOOP 2018 imagery, we used an unsupervised, object‐based classification to classify 2021 winter imagery to be used in measuring interspersion, but only classified water and vegetation. We also used lower spectral and spatial detail (15 and 5, respectively) which was sufficient due to the high contrast generally observed between vegetation and water (with snow being used as a proxy for water). Furthermore, we used a minimum segment size of 180 pixels, thereby allowing for the detection of features of approximately 1 m^2^ or greater.

**TABLE A1:** Index of camera obstruction at Osler Marsh, Ontario.

Value	Description
0	Water virtually clear of plant material and other objects. Muskrats can swim freely.
1	Some plant material but likely no impact on muskrat detection or muskrat movement.
2	Some plant material potentially leading to missed muskrat detections. Muskrat movement may be restricted.
3	Thick plant material potentially leading to missed muskrat detections. Muskrat movement may be restricted.
4	Very thick plant material causing near complete obstruction of viewshed. Muskrat navigation unlikely.

**TABLE A2:** Zero‐inflated negative binomial models of intensity of use by muskrats at Osler Marsh, Ontario.

	Variable	Estimate	Std. error	*z*‐value	*p*
Interspersion + Period					
Count	Interspersion	7.97e^−4^	1.38e^−3^	0.576	0.565
	Period	0.214	0.162	1.32	0.186
Zero‐inflation	Interspersion	−5.74e^−3^	7.11e^−3^	−0.807	0.419
	Period	−0.758	0.473	−1.60	0.109
Interspersion + Water area					
Count	Interspersion	1.68e^−3^	1.38e^−3^	1.22	0.225
	Water area	−6.66e^−4^	4.40e^−4^	−1.51	0.130
Zero‐inflation	Intercept*	−9.48	79.8	−0.119	0.905
Interspersion + Channelization					
Count	Interspersion	−2.24e^−4^	1.30e^−3^	−1.72	0.863
	Channelization	1.08	0.392	2.75	6.03e^−3^
Zero‐inflation	Interspersion	−9.67e^−3^	7.42e^−3^	−1.30	0.192
	Channelization	0.522	1.40	0.374	0.709
Interspersion + Viewshed obstruction					
Count	Interspersion	8.04e^−4^	1.52e^−3^	0.528	0.597
	Viewshed obstruction	−5.62e^−2^	0.226	−0.248	0.804
Zero‐inflation	Interspersion	−8.81e^−3^	5.32e^−3^	−1.66	9.75e^−2^
	Viewshed obstruction	0.965	0.736	1.31	0.189
Interspersion + Sample area					
Count	Interspersion	7.32e^−4^	1.48e^−3^	0.496	0.620
	Sample area	‐2.05e^−2^	0.517	−0.040	0.968
Zero‐inflation	Interspersion	−9.87e^−3^	7.07e^−3^	−1.40	0.162
	Sample area	−1.41	1.72	−0.819	0.413
Interspersion					
Count	Interspersion	9.44e^−4^	1.53e^−3^	0.615	0.538
Zero‐inflation	Interspersion	−1.26e^−2^	1.23e^−2^	−1.02	0.307
Water area					
Count	Water area	−2.09e^−4^	4.46e^−4^	−0.469	0.639
Zero‐inflation	Water area	2.99e^−3^	2.84e^−3^	1.05	0.293
Channelization					
Count	Channelization	1.03	0.434	2.38	1.72e^−2^
Zero‐inflation	Channelization	−0.526	2.38	−0.221	0.825
Sampling period					
Count	Sampling period	0.176	0.157	1.12	0.263
Zero‐inflation	Sampling period	−0.858	0.389	−2.21	2.74e^−2^
Viewshed obstruction					
Count	Viewshed obstruction	−6.67e^−2^	0.228	−0.293	0.770
Zero‐inflation	Viewshed obstruction	12.5	43.5	0.288	0.774
Sampling area					
Count	Sample area	−1.78e^−2^	0.534	−3.30e^−2^	0.973
Zero‐inflation	Sample area	−1.20	1.53	−0.784	0.433
Null model					
Count	Intercept	0.816	0.201	4.06	4.99e^−5^
Zero‐inflation	Intercept	−8.02	88.8	−9.00e^−2^	0.928

* Predictor effects in the zero‐inflation component of the model including interspersion and water area were not identifiable; therefore, zero inflation was modeled using an intercept‐only term.
